# RETRACTED: Di Paola et al. Combined Effects of Potassium Perchlorate and a Neonicotinoid on Zebrafish Larvae (*Danio rerio*). *Toxics* 2022, *10*, 203

**DOI:** 10.3390/toxics13020074

**Published:** 2025-01-22

**Authors:** Davide Di Paola, Fabiano Capparucci, Sabrina Natale, Rosalia Crupi, Salvatore Cuzzocrea, Nunziacarla Spanò, Enrico Gugliandolo, Alessio Filippo Peritore

**Affiliations:** 1 Department of Chemical, Biological, Pharmaceutical and Environmental Science, University of Messina, 98166 Messina, Italy; davide.dipaola@unime.it (D.D.P.); fabiano.capparucci@unime.it (F.C.); sabrina.natale@unime.it (S.N.); aperitore@unime.it (A.F.P.); 2 Department of Veterinary Science, University of Messina, 98168 Messina, Italy; rcrupi@unime.it (R.C.); egugliandolo@unime.it (E.G.); 3 Department of Pharmacological and Physiological Science, School of Medicine, Saint Louis University, Saint Louis, MO 63104, USA; 4 Department of Biomedical and Dental Sciences and Morphofunctional Imaging, University of Messina, 98124 Messina, Italy

The journal retracts the article, “Combined Effects of Potassium Perchlorate and a Neonicotinoid on Zebrafish Larvae (*Danio rerio*)” [1], cited above.

Following publication, concerns were brought to the attention of the Editorial Office regarding image duplications within this publication [1] and between this and an earlier article [2], produced by the same authorship group.

Adhering to our standard procedure, an investigation was conducted by the Editorial Office and the Editorial Board that confirmed duplications within Figure 1, showing different experimental conditions. While the authors collaborated during the investigation, they were unable to satisfactorily explain the image duplication or provide satisfactory raw material for Editorial Board evaluation. Consequently, the Editorial Board has lost confidence in the reliability of the findings and decided to retract this publication [1], as per MDPI’s retraction policy (https://www.mdpi.com/ethics#_bookmark30).

This retraction was approved by the Editor-in Chief of the journal *Toxics*.

The authors did not agree to this retraction.
